# Effectiveness of interventions to improve, maintain or facilitate oral food and/or drink intake in people with dementia: systematic review

**DOI:** 10.1186/1472-6963-14-S2-P1

**Published:** 2014-07-07

**Authors:** Asmaa Abdelhamid, Diane Bunn, Angela Dickinson, Anne Killett, Fiona Poland, John Potter, Kate Richardson, David Smithard, Chris Fox, Lee Hooper

**Affiliations:** 1 University of East Anglia, Norwich, UK; 2 University of Hertfordshire, Hertfordshire, UK; 3 Norfolk and Norwich University Hospital, Norwich, UK; 4 East Kent Hospitals NHS Trust, Kent, UK

## Background

There are over 0.8 million people living with dementia in the UK. The needs of people with dementia are increasingly complex as the illness progresses. Eating and drinking difficulties are a major source of ill health and stress across the stages of dementia in multiple settings.

The evidence on what interventions support people with dementia in continuing to eat and drink well needs to be updated, and the full set of interventions assessed. To ensure people with dementia and their carers have access to the best current evidence, which address the questions that are important to them, we consulted with stakeholder groups and formulated specific questions to be addressed by a systematic review. The evidence will be summarised in light of these questions.

## Materials and methods

The review protocol (http://www.crd.york.ac.uk/ PROSPERO/ DisplayPDF.php?ID= CRD42014007611) was shared with members of two patient and public involvement groups. The members were asked to comment on the protocol and suggest questions they would like the review to address. Compiled questions were added to the protocol to inform our search strategies.

We have conducted a comprehensive search of 13 databases for studies that assess the effectiveness of interventions to improve, maintain or facilitate oral food and drink intake, nutrition and/or hydration status, in people with dementia. Screening studies for eligibility, assessing risk of bias and extracting relevant data is underway and duplicated independently. Studies included in the review will be used to address the specific questions asked.

## Results

The stakeholders’ questions, that were not originally specifically addressed by the review protocol included issues around personalisation of interventions, the relationship with the carer, meaningful activity around food as well as specific issues around oral hygiene and swallowing difficulties. These questions are guiding the way that the review is conducted and will help focus the way that information from the review is provided to people with dementia and their carers.

## Discussion

This systematic review aims to review the effectiveness of interventions to improve, maintain or facilitate oral food and/or drink intake in people with dementia and address specific questions raised by stakeholders. By soliciting and then addressing questions within the systematic review that are important to our stakeholders we will improve the usability of our findings - the questions and their summary answers will be used to inform best practice.

